# Reorganization of the sensorimotor cortex in patients with Ph-negative myeloproliferative neoplasms according to functional MRI

**DOI:** 10.3389/fnins.2025.1677038

**Published:** 2026-01-14

**Authors:** Polina I. Kuznetsova, Sofya N. Morozova, Anton A. Raskurazhev, Marine M. Tanashyan

**Affiliations:** 1 Laboratory of Neuropharmacological fMRI, 1st Neurological Department (Angioneurology), Russian Center of Neurology and Neurosciences, Moscow, Russia; 2 Laboratory of Neuropharmacological fMRI, Radiology Department, Russian Center of Neurology and Neurosciences, Moscow, Russia

**Keywords:** cerebrovascular disease, functional brain MRI, motor cortex reorganization, sensorimotor cortex reorganization, Ph-negative myeloproliferative neoplasms, neurovascular unit

## Abstract

**Introduction:**

Myeloproliferative neoplasms (MPN) may contribute to cerebrovascular disease via cellular and endothelial pathology leading to impairment at the neurovascular unit (NVU) level. Studies targeting this patient cohort form a neuroscientific viewpoint are scarce.

**Objective:**

We aimed at elucidating possible neuroimaging correlates of NVU alterations in MPNs patients.

**Materials and methods:**

We initially included 187 patients with MPNs in this study, retaining 39 patients as per eligibility criteria (25.6% males, median age – 43 years), who were matched with a control group of 11 healthy subjects (36.4% males, median age – 41 years). Structural and task-based (motor paradigm) functional MRI were performed in both groups, along with the evaluation of baseline blood parameters (hemoglobin, hematocrit and platelet count), comorbidities (arterial hypertension, diabetes mellitus, atherosclerosis) and antiplatelet use: these factors were then used as covariates in statistical analysis.

**Results:**

fMRI data analysis in the group of MPN patients revealed activation in the left primary sensorimotor cortex (pre- and post-central gyri); the right supramarginal gyrus showed significant activation (*T* = 5.99, p_FWEcorr_ = 0.015) in the MPN group only. Group fMRI data analysis in healthy volunteers showed two main clusters of activation in the left precentral gyrus and right hemisphere of the cerebellum during task execution. Second-level analysis of activation differences between MPN patients and healthy volunteers showed greater activation in the right primary sensorimotor cortex in MPN (Puncorr = 0.014 and <0.001 at cluster and peak level respectively).

**Conclusion:**

Additional task-specific cortical activation in MPN patients may be potentially linked to NVU disturbance, even in otherwise unchanged cerebral activation patterns. Our findings also suggest that fMRI data in MPN may be confounded by higher blood cell count that needs to be controlled for in this cohort of patients.

## 1 Introduction

Myeloproliferative neoplasms (MPN) are a rare but significant cause of cerebrovascular pathology due to high risk of thrombohemorrhagic events (i.e., stroke) (Tanashyan et al., 2024). Ph-negative MPN include three common diseases: polycythemia vera (PV), essential thrombocythemia (ET), and primary myelofibrosis (PMF). The pathogenesis of these disorders is based on genetic mutations in genes such as *JAK2*, *CALR*, and *MPL*, leading to clonal hematopoiesis and qualitative and quantitative changes in blood cells (Gerds et al., 2022). The latter increase the risk of arterial and venous thrombosis (Todor et al., 2024). The overall 5-year risk of vascular disease ranges from 0.5 to 7.7% in patients with MPN, which is higher than the risk in the general population (Frederiksen et al., 2019). Despite advances in the diagnosis and treatment of MPN, vascular complications continue to be a major cause of worsening prognosis and reduced quality of life for patients (Kuznetsova et al., 2022). This is due to the complex nature of the problem, which is determined by a variety of factors, including hypercoagulation, endothelial dysfunction, systemic inflammation, and dysregulation of blood cell production and microcirculation (Farina et al., 2021). At the molecular and cellular levels, these processes are intricately linked and require a thorough investigation using modern translational approaches that combine clinical data and novel neuroimaging techniques.

Given the systemic and multifactorial impact of MPN on cerebral circulation, there is a compelling rationale to explore brain functional alterations beyond gross vascular events. In this context, functional magnetic resonance imaging (fMRI) emerges as a noninvasive tool capable of detecting subtle changes in neurovascular function and brain network dynamics. Of particular interest is the assessment of the neurovascular unit (NVU) — the integrated complex of neurons, glial cells, pericytes, and endothelial cells that regulates cerebral blood flow in response to neuronal activity (Lecrux and Hamel, 2011; Costantino Iadecola, 2004). Disruption of the NVU has been increasingly recognized as a pivotal mechanism underlying cerebrovascular pathology and cognitive impairment in various systemic and central nervous system diseases. In patients with MPN, chronic inflammation, endothelial injury, and thrombosis may impair the NVU’s ability to appropriately modulate cerebral perfusion, which can precede and potentiate overt ischemic injury (Bhuria et al., 2022). Functional MRI, through blood oxygenation level-dependent (BOLD) signal changes and advanced connectivity analyses, offers the capability to quantify neurovascular coupling efficiency, identify regions of altered hemodynamics, and elucidate compensatory brain network adaptations (Zhang et al., 2025).

Integration of fMRI-derived biomarkers with clinical and laboratory parameters provides a translational framework to characterize the impact of MPN-driven vascular alterations of brain function. This approach promises to enhance early detection of cerebral involvement for vascular events and guide targeted therapeutic interventions aimed at preserving neurovascular integrity and preventing stroke-related morbidity in this vulnerable population.

## 2 Materials and methods

### 2.1 Study population and clinical data

**Figure 1:**
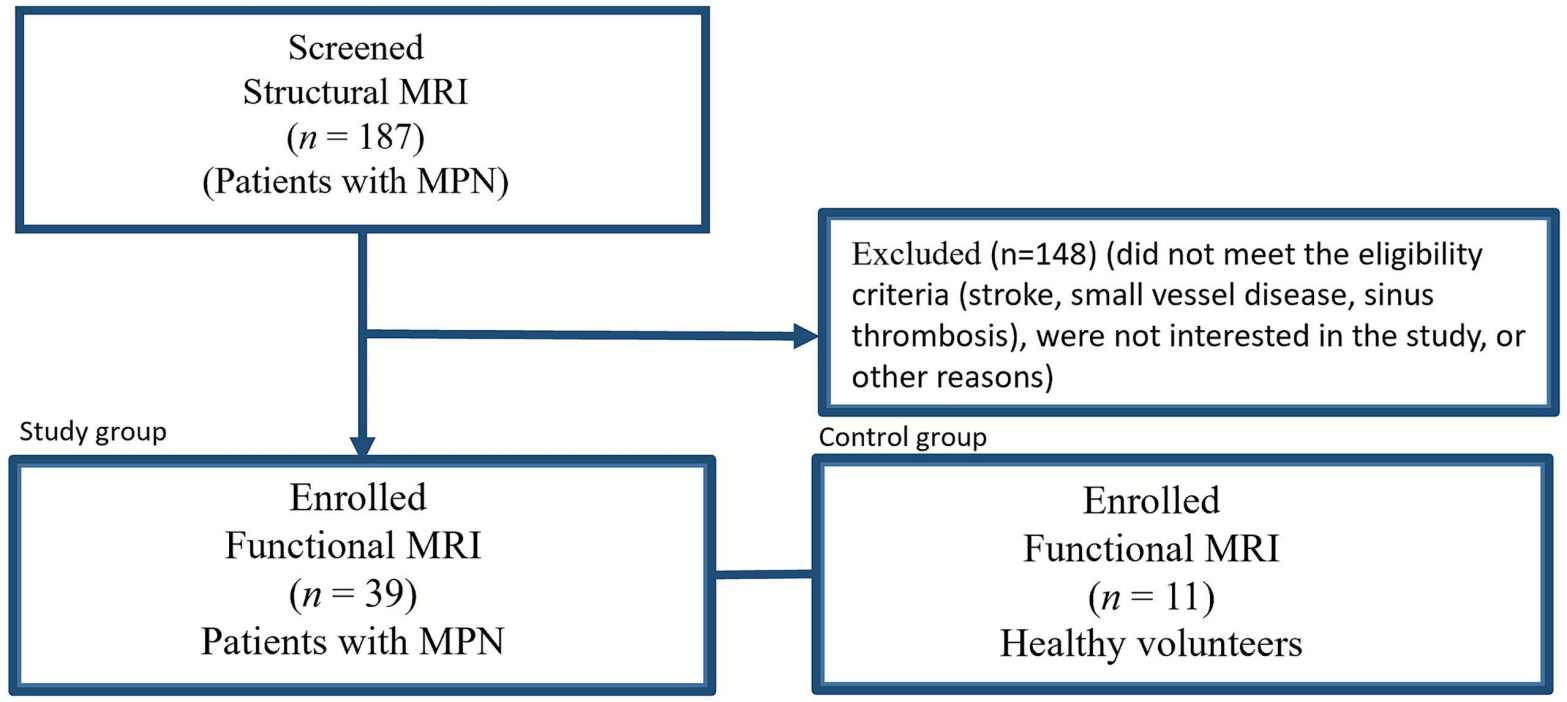
Flowchart. The formation of a study group and a control group.

The study was conducted on the basis of the «Russian Сenter of Neurology and Neurosciences» from November 2022 to April 2025. 187 patients with an established diagnosis of MPN were included in the study (according to WHO criteria, 2016). To verify the diagnosis, data from clinical examination, general blood analysis, trepanobiopsy, and molecular genetic studies were used, including the determination of mutations in the *JAK2* (V617F), *MPL*, *CALR*, and *BCR*/*ABL1* genes (National Medical Research Center for Hematology). The inclusion criteria were an established diagnosis of one of the nosological forms of Ph-negative MPN, signed informed consent, and absence of post-stroke lesions according to conventional magnetic resonance imaging (MRI). The exclusion criteria were the lack of consent, the presence of severe concomitant somatic pathology, acute/massive stroke, small vessel disease, tumors, inflammatory and autoimmune diseases, contraindications to MRI. All patients underwent detailed clinical and neurological examination, laboratory analyses: complete blood count (hemoglobin, platelet count, hematocrit). All patients with MPN underwent conventional brain structural magnetic resonance imaging (MRI). The study sample was formed by continuous inclusion of patients (*n* = 39) who gave consent for further analysis (Figure 1). The control group included healthy volunteers (HV) matched by gender and age, who were not diagnosed with MPN at the time of the study, had no abnormalities in blood tests, and showed no signs of brain damage according to structural MRI. All participants have given informed consent.

### 2.2 Laboratory analysis

A complete blood count (CBC) was performed using an automated hematology analyser. Venous blood samples were collected under fasting conditions into vacuum tubes containing ethylenediaminetetraacetic acid (EDTA) as an anticoagulant. All samples were processed and analysed within 2 h of collection. The following parameters were assessed: hemoglobin concentration (Hb, g/L), platelet count (PLT, ×10^9^/L) (performed by automated counting), hematocrit (Hct, %).

### 2.3 Statistical analysis

Statistical analysis and visualization were performed using the jaMovi software (v. 2.6). Descriptive statistics were used to summarize demographic and laboratory parameters, with results reported as median and interquartile ranges (Me [Q1; Q3]) for continuous variables, and as counts and percentages for categorical variables. Comparisons of continuous variables (such as age, hemoglobin, platelet count, hematocrit) between patients with MPN and healthy volunteers (HV) were conducted using the Mann–Whitney U test, given the small sample size and non-Gaussian data distribution. Comparison of categorical variables (such as gender, presence of arterial hypertension, diabetes mellitus, history of thrombosis, carotid atherosclerosis, and presence of *JAK2* V617F mutation) were assessed using Fisher’s exact test. Statistical significance was set at a two-sided *p*-value < 0.05.

### 2.4 MRI data acquisition and processing

MRI data were acquired from 39 patients and 11 healthy control subjects using Siemens MAGNETOM Prisma 3 T scanner with a following protocol. Conventional MRI sequences were used to evaluate brain tissue and exclude substantial concomitant brain pathology (acute/massive stroke, tumors, inflammatory and autoimmune diseases), which was the exclusion criterion for the study: axial T2 (TR = 5,500 ms, TE = 106 ms, FOV = 220 mm, in-plane resolution 0.7 × 0.7 mm, slice thickness = 3 mm, distance factor 30%, number of slices = 35, acquisition time = 2 min 14 s), axial T2FLAIR (TR = 9,000 ms, TE = 88 ms, FOV = 220 mm, in-plane resolution 0.4 × 0.4 mm, slice thickness = 4 mm, number of slices = 27, distance factor 30%, acquisition time = 1 min 50 s), axial SWI (TR = 31 ms, TE = 20 ms, FOV = 260 mm, in-plane resolution 1.0 × 1.0 mm, slice thickness = 1.5 mm, number of slices = 80, distance factor 20%, acquisition time = 5 min 37 s), DWI (TR = 3,700 ms, TE = 55 ms, FOV = 220 mm, in-plane resolution 1.1 × 1.1 mm, slice thickness = 4 mm, number of slices = 27, distance factor 30%, b factor 0 and 1,000, acquisition time = 1 min 41 s) and sagittal T1MPRAGE isometric sequence to obtain anatomical data (TR = 2,300 ms, TE = 2.98 ms, FOV = 256 mm, slice thickness = 1 mm, in-plane resolution 1 × 1 mm, number of slices = 176, acquisition time = 5 min 12 s). Functional MRI was performed using an axial echo-planar gradient echo sequence with block motor paradigm (task fMRI) (TR 2000 ms, TE 21 ms, number of slices 44, distance factor 25%, slice thickness 2 mm, in-plane resolution 2 × 2 mm, FOV = 192 mm, flip angle 70 degrees, 150 measurements: 5 baseline blocks of 15 measurements, 5 blocks with a paradigm of 15 measurements, each block duration 30 s). During the paradigm blocks, the patient’s screen displayed alternating still (baseline blocks) and pulsing with 0.5 Hz frequency heart (active blocks). Patients and HV were given the task to squeeze small rubber ball by their right hand with the same frequency as the heart pulsing. All patients and healthy subjects were trained before examination outside the scanner.

### 2.5 Task fMRI pre- and post-processing

Task fMRI data were processed using the SPM12 software package based on MATLAB R2022a. After standard preprocessing including spatial reorientation, co-registration, normalization to MNI stereotaxic space and smoothing, first-level fMRI data analysis was performed and activation areas were obtained for each subject both as graphic color maps superimposed on anatomical data and as digital data including level of statistical significance, volume and coordinates of activation area in the MNI stereotaxic space (Fonov et al., 2009). This analysis was performed for each subject separately, followed by a group analysis: multiple regression design was used with a threshold of statistical significance - *p* ≤ 0.05, the effect size of multiple comparisons was assessed by family-wise error (FWE), *T* > 4.5. For between group analysis, two-sample t-test was used with a statistical significance threshold of *p* ≤ 0.05 with correction for multiple comparisons. Multiple covariates including hemoglobin level, platelet count, hematocrit level, the presence of arterial hypertension, atherosclerosis, diabetes mellitus, and use of antiplatelet agents were included as potentially viable confounders. This approach allowed controlling for the influence of these factors, thereby improving the validity and interpretability of the results obtained from the functional imaging data. Subsequently, xjView 9.0 (Human Neuroimaging Lab, Baylor College of Medicine) based on SPM12, was used to localize areas of interest, view and present the obtained data.

## 3 Results

Participant demographics and clinical data are listed in Table 1.

**Table 1:** Participant demographics and clinical data.

Clinical parameters	MPN (*n* = 39) 24%	HV (*n* = 11)	*p* < 0.001
Men, *n* (%)	10 (25.6%)	4 (36.4%)	0.484
Age, Me [Q1; Q3]	43 [37; 52]	41 [28; 50]	0.582
Hemoglobin (g/l), Me [Q1; Q3]	136 [129; 154]	142 [119; 148]	0.367
Platelet (х10^9^), Me [Q1; Q3]	452 [269; 621]	258 [177; 510]	0.012
Ht, Me [Q1; Q3]	41 (38; 45)	42 (35; 44)	0.543
ET, *n* (%)	15 (38.5%)	NA	NA
PV, *n* (%)	24 (61.5%)	NA	NA
Arterial hypertension, *n* (%)	14 (39)	4 (36)	0.976
Diabetes mellitus, *n* (%)	3 (8)	0 (0)	NA
History of venous thrombosis, *n* (%)	3 (7)	0 (0)	NA
*JAK2* V617F, *n* (%)	30 (77)	0 (0)	NA
Cytoreductive therapy, *n* (%)	20 (51)	0 (0)	NA
Antiplatelet agents, *n* (%)	39 (100)	0 (0)	NA
Carotid atherosclerosis, *n* (%)	9 (23)	2 (18)	0.726

MPN, myeloproliferative neoplasms; HV, healthy volunteers; Ht, hematocrit; ET, essential thrombocythemia; PV, polycythemia vera.

There were no statistically significant differences between the MPN and HV groups in terms of gender distribution (25.6% males in MPN vs. 36.4% in HV, *p* = 0.484) or median age (43 years [37; 52] vs. 41 years [28; 50], *p* = 0.582). Hemoglobin levels were comparable between groups (median 136 g/L [129; 154] in MPN vs. 142 g/L [119; 148] in HV, *p* = 0.367). Hematocrit values showed no significant difference (41% [38; 45] vs. 42% [35, 44], *p* = 0.543). A statistically significant elevation in platelet count was observed in the MPN group (452 × 10^9^/L [269, 621]) compared to healthy volunteers (258 × 10^9^/L [177, 510]), with *p* = 0.012. This reflects the characteristic thrombocytosis associated with MPN. Conditions such as arterial hypertension (39% in MPN vs. 36% in HV), diabetes mellitus (8% vs. 0%), history of venous thrombosis (7% vs. 0%), and carotid atherosclerosis (23% vs. 18%) indicate a higher burden of cardiovascular comorbidities in patients with myeloproliferative neoplasms (MPNs) compared to otherwise healthy controls. This elevated prevalence of vascular risk factors can have important implications for cerebral hemodynamics and overall neurovascular health, potentially influencing the results of functional neuroimaging studies. All MPN patients took antiplatelet agents, while none received antithrombotics in the control group.

**Figure 2:**
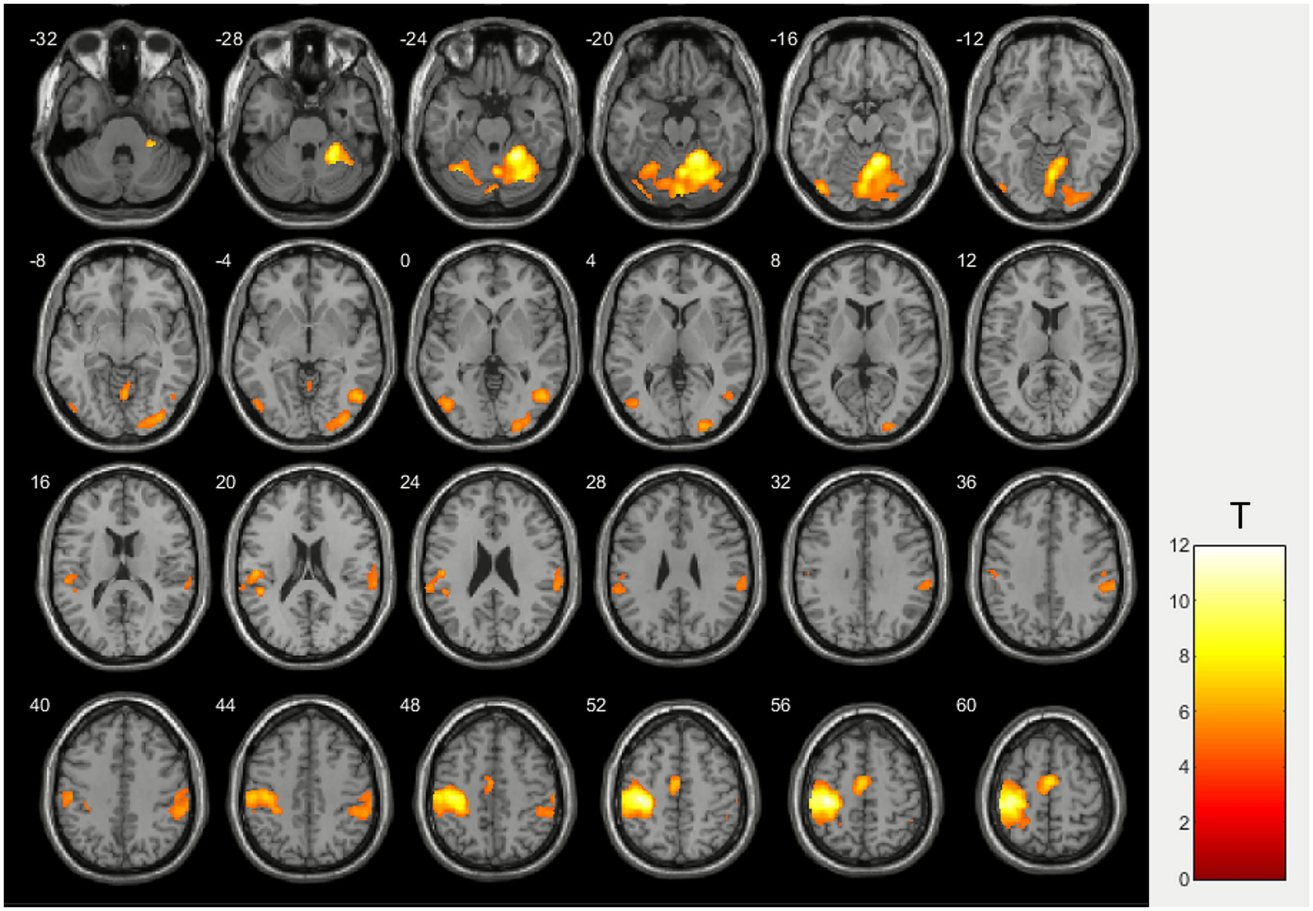
Group level right hand ball squeezing activation for patients with MPN.

Group fMRI data analysis in the MPN group showed activation in the left sensorimotor cortex (pre- and post-central gyri) spreading to the left supramarginal gyrus and also in the right supramarginal gyrus, supplementary motor area and right hemisphere of the cerebellum, spreading to the left hemishere (Figure 2). Activation peaks coordinates and statistical data are presented in Table 2.

**Table 2:** Activation clusters details for patients with MPN during squeezing a ball with the right hand.

Activation cluster	Activation peak coordinates in the MNI space	Т*	p_FWEcorr_
Right hemisphere of the cerebellum	24 −48 −24	10.9	0.000
Left sensorimotor cortex on the left (pre- and post-central gyri)	−36 −24 54	11.9	0.000
Right supramarginal gyrus	58 −36 40	5.99	0.015
Supplementary motor area	2 −6 60	7.9	0.000

**Figure 3:**
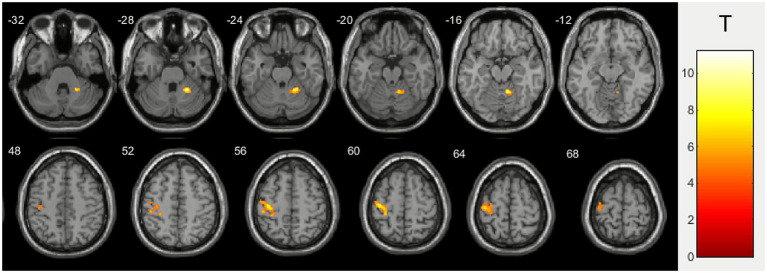
Group level right hand ball squeezing activation for healthy volunteers.

Group fMRI data analysis in healthy volunteers showed two main clusters of activation in the left precentral gyrus and right hemisphere of the cerebellum during right hand ball squeezing (Figure 3). Activation peak coordinates and statistical data are presented in Table 3.

**Table 3:** Activation clusters details for healthy volunteers during squeezing a ball with the right hand.

Activation cluster	Activation peak coordinates in the MNI space	Т*	p_FWEcorr_
Right hemisphere of the cerebellum	24 −48 −26	11.2	0.001
Left precentral gyrus	−38 −20 58	8.4	0.000

**Figure 4:**
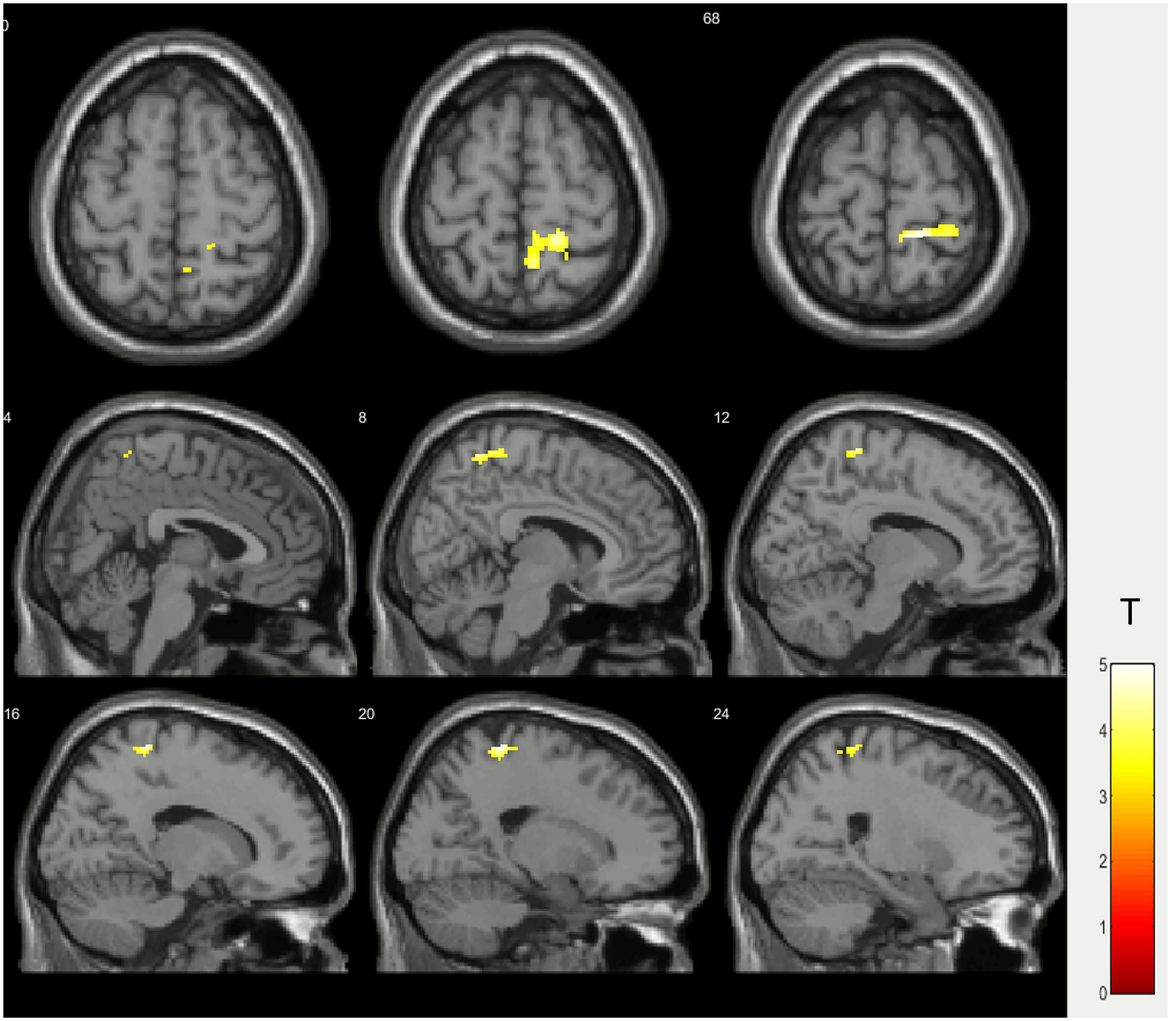
Second-level group activation difference (two-sample *t*-test) between patients with myeloproliferative neoplasms and healthy volunteers (MPN > normal).

Second-level analysis of activation differences between MPN patients and healthy volunteers group showed higher activation in the right primary sensorimotor cortex in the study group during motor task (Figure 4; Table 4).

**Table 4:** Activation differences details during right hand ball squeezing between the group of patients with myeloproliferative diseases and the control group (MPN > normal).

Activation cluster	Activation peak coordinates in the MNI space	Т*	p_FWEcorr_ (cluster-level)	p_uncorr_ (cluster-level)	p_FWEcorr_ (peak-level)	p_uncorr_ (peak-level)
Right primary sensorimotor cortex	18 −40 66	4.99	0.104	0.014	0.137	0.000

*T is the critical value of the Student’s *t*-distribution, an indicator of the reliability of a statistical hypothesis. It is recommended for use in samples with less than 30 individuals, when the standard deviation of the population is unknown.

## 4 Discussion

In this study, task-based functional magnetic resonance imaging (fMRI) was used to assess brain activation during a standard motor task (right-hand ball squeezing) in patients with myeloproliferative neoplasms (MPN) and healthy volunteers (HV). The group analyses demonstrated both shared and distinct patterns of activation, providing new insights into the neural mechanisms underlying motor function in MPN and highlighting the complex interplay between hematological abnormalities and neurovascular responses. In both MPN patients and healthy controls, the task robustly activated classical motor circuitry: primary sensorimotor cortex (pre- and post-central gyri) in the left hemisphere (contralateral to the performing hand) showed the highest activation in both groups (*T* = 11.9 and 8.4, p_FWEcorr_ = 0.000), consistent with well-established models of somatotopic organization and voluntary movement control (Yousry et al., 1997). The right cerebellum (ipsilateral to performing hand) exhibited significant activation (*T* > 10, p_FWEcorr_ < 0.001 in both groups), which is consistent with its established involvement in motor coordination and cognitive functions (King et al., 2019; Stoodley et al., 2012; Klein et al., 2016). This activation may also reflect potential compensatory mechanisms, particularly in relation to right-hand movements mediated through crossed cerebro-cerebellar networks (Buckner et al., 2011). The supplementary motor area (SMA) showed significant recruitment in MPN patients (*T* = 7.9, p_FWEcorr_ = 0.000), which may reflect an increased demand for motor planning and initiation or a possible compensatory response to subtle dysfunctions within cortico-cerebellar integration networks (Ramnani, 2012; Schulz et al., 2017).

Interestingly, significant activation of the right supramarginal gyrus (SMG) (*T* = 5.99, p_FWEcorr_ = 0.015) was observed exclusively in the MPNs group. The SMG is known to be involved in sensorimotor integration, somatosensory attention, and higher-order motor control (Peng et al., 2024), which may suggest that patients with MPN engage these secondary integrative circuits to a greater extent. This pattern of activation might reflect a compensatory response to subtle impairments in primary motor pathways or alterations in sensorimotor feedback potentially related to vascular, microcirculatory, or inflammatory changes associated with the disease (Ben-Shabat et al., 2015).

As for the between-group difference: the comparison analysis (MPNs > controls) revealed significantly greater activation in the right primary sensorimotor cortex in the patient group (*T* = 4.99). In second-level between group analysis statistically significant activation was greater in the right primary sensorimotor cortex (p_uncorr_ = 0.014 and <0.001 at cluster and peak level respectively), yet these differences were found to be non-significant after multiple comparison correction (p_FWE-corr_ = 0.104 and 0.137 respectively). The relatively low significance levels observed after correction for multiple comparisons may be influenced by the limited sample size of the comparison groups. Small sample sizes inherently reduce statistical power in fMRI studies, potentially limiting the ability to detect true effects at a stringent correction level. Therefore, these findings should be interpreted with caution, and future studies with larger cohorts are warranted to confirm and extend our results. Increased activation in the ipsilateral sensorimotor cortex may represent a compensatory mechanism. In patients with neurological dysfunction or injury, it is common to observe enhanced engagement of motor areas in the hemisphere ipsilateral to limb movement (usually considered non-dominant for that task) as the brain reorganizes to maintain motor function (Andrushko et al., 2022). This compensatory recruitment may reflect adaptive neuroplasticity supporting precise motor performance despite underlying pathology (Kong et al., 2020).

In summary, the findings highlight a trend toward increased sensorimotor cortex activation in patients with MPN, potentially indicating compensatory cortical reorganization, but the statistical non-significance after correction underscores the need for cautious interpretation and further studies with larger sample sizes. The observed neuroimaging patterns may be caused by subtle microvascular impairment or neurovascular unit (NVU) dysfunction associated with MPN (Schafer, 2021). The term NVU was first coined at the 2001 Stroke Progress Review Group meeting of the National Institute of Neurological Disorders and Stroke. The NVU generally consists of endothelial cells, pericytes, astrocytes, basal lamina, neurons, microglia, and circulating blood flow (Iadecola, 2017). The concept of neurovascular coupling refers to the process by which a transient neural activity triggers a corresponding increase in cerebral blood flow. The advancement of various functional neuroimaging techniques for assessing cerebral functions *in vivo* is at least partly dependent on this phenomenon (Huneau et al., 2015).

Importantly, when performing fMRI statistical analysis we opted to include as confounding factors (covariates) routine blood-based markers (i.e., platelet count, hemoglobin, hematocrit) as well as certain clinical determinants (e.g., arterial hypertension, diabetes, etc.) to account for factors that may directly influence BOLD signal and neurovascular coupling (especially in MPN). This methodological approach, at least partially, may remove a certain amount of signal fluctuations and reveal implicated neural and neurovascular changes.

In a study by Ward et al. (2020) on a significant cohort of healthy subjects (518 people), it was shown that hemoglobin levels significantly affect global and local indicators of functional connectivity via fMRI. Individual hemoglobin differences may be a covert confounder in fMRI analyses – both task- and rest-state. Even subtle (i.e., within reference value range) alterations in hemoglobin and/or hematocrit may influence quantitative indicators in functional neuroimaging. Ward and colleagues suggest that accounting for hemoglobin/hematocrit levels may be a viable strategy in connectome studies (Ward et al., 2020).

In our study fMRI data shows differences in brain activity between patients with MPN and healthy volunteers. These changes reflect the potential involvement of the neurovascular unit and its role in cerebral circulation dysfunction associated with MPN. The latter may be of special importance as functional changes may precede overt cerebrovascular disease, offering time and incentive for targeted prevention. These findings collectively suggest that while the overall motor activation pattern is preserved in MPN patients, there is evidence of increased and redistributed engagement of integrative motor and sensorimotor regions.

Future research in this area should focus on identifying the potential prognostic value of early subclinical changes in MPN (e.g., stroke and/or white matter lesions in longitudinal studies); implementing other important pathogenetic biomarkers (e.g., inflammation, RBC deformability); obtaining potential reference fMRI-patterns to use in personalized approaches.

## 5 Conclusion

Patients with myeloproliferative neoplasms demonstrate both preserved and altered patterns of brain activation during simple motor tasks. Increased activation in specific motor regions, including the supramarginal gyrus and paracentral lobule, may reflect adaptive neural responses to underlying neurovascular and microcirculatory impairments. Albeit statistically non-significant, greater activation of the ipsilateral primary sensorimotor cortex in MPN (vs healthy volunteers) may corroborate the presence of altered neuroimaging pattern in this group of patients. These findings highlight the potential of fMRI to detect subtle, clinically covert changes in brain function in patients with blood disorders, particularly when considering confounding factors such as hematological conditions.

### 5.1 Limitations

The study has several limitations, of which the clinical heterogeneity of the study group and relatively small sample size of the control group may be the most important. Myeloproliferative neoplasms (MPN) encompass several distinct entities (PV, ET, etc.), each potentially associated with different vascular, inflammatory, and neurocognitive profiles. The grouping of all MPN patients together could conceal disorder-specific patterns or mechanisms in neural activation. Although covariates like platelet count, hemoglobin, and hematocrit were included to control their influence on the BOLD signal in healthy volunteers, residual and unmeasured effects of abnormal blood rheology and oxygenation in MPN patients could directly affect BOLD responsiveness and neurovascular coupling, possibly confounding true neural activation patterns. Also, we implied a linear relationship between included covariates and fMRI signal intensity which may not be the case and needs further reappraisal in cohorts of patients with extreme (both high- and low) values of hemoglobin and hematocrit. One more important limitation of our study is the potential bias introduced by the fact that all patients with myeloproliferative neoplasms were receiving concurrent antiplatelet therapy. This pharmacological intervention could have influenced neurophysiological parameters and functional brain activation patterns observed in our research. It is noteworthy, however, that our study included a healthy control group that did not receive any antithrombotic or antiplatelet treatment, which allows some degree of comparative assessment. Nevertheless, the presence of antiplatelet therapy in all patients with MPN remains a confounding factor that should be taken into account when interpreting the results, and represents a limitation that may affect the generalizability of our findings. Further studies controlling for or stratifying by antithrombotic medication status are warranted to clarify these effects.

While the study provides valuable insights into altered brain network recruitment in MPN, the interpretation of results is constrained by sample size, heterogeneity, potential confounding from blood physiology, and lack of multimodal or functional validation. Future work should aim for larger, more homogeneous cohorts, multimodal imaging, longitudinal follow-up, and integration of clinical outcomes to better understand the neurological consequences of MPN.

## Data Availability

The raw data supporting the conclusions of this article will be made available by the authors, without undue reservation.
